# Revisit the Cellular Transmission and Emerging Techniques in Understanding the Mechanisms of Proteinopathies

**DOI:** 10.3389/fnins.2021.781722

**Published:** 2021-11-18

**Authors:** Jinwen Jiang, Yu Liu, Qihui Wu

**Affiliations:** Translational Research Institute of Brain and Brain-Like Intelligence, Shanghai Fourth People’s Hospital Affiliated to Tongji University School of Medicine, Shanghai, China

**Keywords:** Alzheimer’s disease, Parkinson’s disease, Aβ cascade hypothesis, α-synuclein aggregation and spreading, transcriptomics of nervous system

## Abstract

Alzheimer’s and Parkinson’s diseases (AD and PD) are amongst top of the prevalent neurodegenerative disease. One-third of PD patients are diagnosed with dementia, a pre-symptom of AD, but the underlying mechanism is elusive. Amyloid beta (Aβ) and α-synuclein are two of the most investigated proteins, whose pathological aggregation and spreading are crucial to the pathogenesis of AD and PD, respectively. Transcriptomic studies of the mammalian central nervous system shed light on gene expression profiles at molecular levels, regarding the complexity of neuronal morphologies and electrophysiological inputs/outputs. In the last decade, the booming of the single-cell RNA sequencing technique helped to understand gene expression patterns, alternative splicing, novel transcripts, and signal pathways in the nervous system at single-cell levels, providing insight for molecular taxonomy and mechanistic targets of the degenerative nervous system. Here, we re-visited the cell-cell transmission mechanisms of Aβ and α-synuclein in mediating disease propagation, and summarized recent single-cell transcriptome sequencing from different perspectives and discussed its understanding of neurodegenerative diseases.

## Introduction

The deposition of β-sheet containing amyloid aggregates featured many neurodegenerative diseases, such as amyloid-β (Aβ) and tau in Alzheimer’s disease (AD), α-synuclein in Parkinson’s disease (PD), and TAR DNA-binding protein (TDP) 43 in amyotrophic lateral sclerosis (ALS), which causes inflammation, neuronal dysfunction, movement and cognition defects. These infectious amyloidogenic “seeds” are self-template dependent replication, transcellular propagation, and transmissible neuropathology between distinct brain regions. An important contribution to the neuropathology is played by the accumulation of unfolded or misfolded proteins leading to the formation of disordered (amorphous) or ordered (amyloid fibril) aggregates, so these diseases are often called “conformational diseases” (Surguchev and Surguchov, 2010). This review discussed the contribution of transcellular propagation of Aβ oligomers/fibrils in AD and α-synuclein (αSyn) aggregates in PD, in which 30–40% of PD has been diagnosed with dementia, an early symptom of AD. It also discussed the recent advance of RNA sequencing techniques in the application of neurodegenerative diseases. Unlike traditional biochemistry and molecular biology, single-cell RNA sequencing (scRNA-seq) helps to understand multiple gene expression patterns and signal pathways in the nervous system at the single-cell level, aiming to impact the development of new diagnostic and therapeutic strategies in the degenerative nervous system.

### The Pathogenesis of AD-Related β-Amyloid Cascade Hypothesis

Alzheimer’s disease is the most common degenerative disease of the nervous system that occurs in the elderly. Its clinical manifestations are mainly characterized by progressive memory decline and later comprehensive cognitive dysfunction. AD patients have a long course of the disease, gradually lose the ability to live, and eventually die of complications due to the inability to take care of themselves (Tokuchi et al., 2016). The two primary cardinal lesions associated with AD are the neurofibrillary tangle and the senile plaque (Perl, 2010). The neurofibrillary tangle consists of abnormal accumulations of phosphorylated tau within the perikaryal cytoplasm of specific neurons. The senile plaque consists of a central core of beta-amyloid, a 4-kD peptide, surrounded by abnormally configured neuronal processes or neurites. Other neuropathological lesions are encountered in cases of AD, but the disease is defined and recognized by these two cardinal lesions. Other lesions include poorly understood changes such as granulovacuolar degeneration and eosinophilic rodlike bodies (Hirano bodies). The loss of synaptic components is a change that has a significant impact on cognitive function and represents another crucial morphological alteration. It is essential to recognize that distinguishing between AD, especially in its early stages, and normal aging may be complicated, particularly if one examines the brains of patients who died at an advanced old age. It is also noted that instances of pure forms of AD, in the absence of other coexistent brain disease processes, such as infarctions or PD-related lesions, are relatively uncommon.

The gross pathological manifestation of AD is brain atrophy (Rabinovici et al., 2016). The etiology and pathogenesis of AD are complex, and there is currently no effective prediction method. Previous clinical and basic research evidence around Aβ pointed out that it played a vital role in the course of AD (Selkoe and Hardy, 2016). However, none of the drugs targeting Aβ passed clinical trials, suggesting the complexity of the onset of AD. The Aβ cascade hypothesis has also been challenged. Re-recognizing the role of the Aβ cascade hypothesis in the pathogenesis of AD and exploring possible new ways of AD will be the direction of future AD research. Here, we summarized the Aβ cascade hypothesis and related AD pathology, pathophysiological process, and treatment research progress.

At present, the pathogenesis and etiology of AD are still unclear. It believes that Aβ is a polypeptide obtained by continuous hydrolysis of amyloid precursor protein (APP) under the action of β and γ secretases. The matrix precipitates and accumulates. Among them, the soluble aggregates of Aβ are considered the main form of neurotoxicity and eventually cause cognitive dysfunction (Sharma et al., 2016) and play a crucial pathological role in AD. The deposition of Aβ aggregates leads to the formation of a large number of SP in the brain.

Aβ directly acts on neurons and produces toxic effects. Moreover, it also activates microglia and astrocytes in the brain to produce cytokines, induce inflammatory response, and trigger cell death eventually. Within the chronic inflammation of the nervous system, activated microglia can engulf synapses in the early stage of AD (Goedert, 2015). In addition, the hyperphosphorylation of the microtubule-associated protein Tau in neurons can also be downstream of the excessive production and aggregation of Aβ, which in turn leads to neuronal degeneration in the brain, leading to AD-related dementia (Pooler et al., 2015).

Misfolding and aggregation of Aβ in the brain is a critical step in the course of AD (Minjarez et al., 2016). Aβ can exist in the brain in three soluble forms, including monomeric Aβ, Aβ oligomers, and fibrillar (Selkoe and Hardy, 2016). Fibrillar Aβ, also known as Aβ fibrils, can act as a pro-oxidant, induce protein oxidation, and change the activity of oxidation-sensitive enzymes (Tycko, 2016). The oligomerized Aβ can be embedded in the bilayer of the neuronal membrane to increase the membrane’s permeability, leading to an increase in reactive oxygen species in the cell, triggering mitochondrial dysfunction, and inducing lipid peroxidation. Therefore, in neurodegenerative diseases related to Aβ, the change of cell membrane permeability is a common phenomenon. In addition, more and more evidence has suggested that Aβ oligomers are the real “culprit” of AD in recent years. After Walsh et al. (2002) reported in 2002 that Aβ oligomers had a damaging effect on rats’ long-term potentiation (LTP), scientists gradually revealed that Aβ aggregation has deleterious effects on neuronal structure and cognition (Tsai et al., 2004; Cleary et al., 2005; Grutzendler et al., 2007). Shankar et al. (2008), extracted Aβ oligomers from the cerebral cortex of AD patients, added them to the hippocampal slice incubation solution, or injected them into the rat lateral ventricle, which caused the attenuation of hippocampal LTP and enhancement of long-term depression (LTD), decreased the hippocampal dendritic spine density and the decline of learning and memory ability in normal rats. This work has consolidated the critical contribution of Aβ oligomers in the pathogenesis of AD from a pre-clinical perspective.

In the normal brain, Aβ is not useless. There is experimental evidence that Aβ monomer has neurotrophic effects (Shin et al., 2015). When the brain is in a physiological state, the production and degradation of Aβ is a dynamic equilibrium process. If too much production or degradation slows down, Aβ will accumulate progressively and become an insoluble form of dense plaque, that is, SP. The removal of SP may increase the content of Aβ oligomers and accelerate neuronal degeneration (Bachhuber et al., 2015; Shi et al., 2017).

**FIGURE 1:**
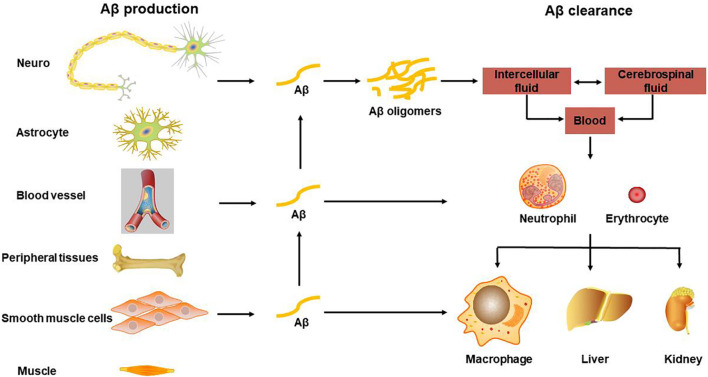
Transport and clearance of Aβ in brain and peripheral tissues and organs. Aβ is produced by neurons and glial cells in the brain, as well as peripheral blood vessel wall cells, skeletal muscle, and osteoblasts, etc., and secreted out of the cell; Aβ in the central nervous system is transported through brain tissue fluid, cerebrospinal fluid, and blood-brain barrier to the peripheral blood; monocytes and neutrophils swallow part of Aβ, some are degraded by Aβ degrading enzymes, and the other part is swallowed by macrophages in peripheral organs and tissues, or is excreted through bile and urine.

After the Aβ in the brain is produced by APP hydrolysis and metabolism, it can be degraded in the brain by glial cells and some enzymes, such as NEP and insulin degradation enzyme (IDE), and it can also be transported through the blood. Aβ is produced from the cell and secreted into the intercellular fluid, enters the cerebrospinal fluid through the brain-cerebrospinal fluid barrier, and transports from the cerebrospinal fluid to the blood and lymphatic system through the blood-brain barrier (BBB) and arachnoid villi or perivascular space (PVS), where the Aβ is cleared but also accumulated within the whole body. The lymphatic system may also pass through the lymphatic vessels of the meninges (Xiang et al., 2015). Through the BBB the transported Aβ is mediated by low-density lipoprotein receptor-related protein 1 (LRP1) (Pflanzner et al., 2011), and the transport from brain tissue fluid to cerebrospinal fluid is through the glial lymphatic system, which is a network of perivascular space and the foot processes of astrocytes surround PVS. There is clinical evidence that decreased cerebral blood flow increases the accumulation of Aβ in the brain (Mattsson et al., 2014), especially Aβ deposited in cerebral blood vessels may be one of the causes of inflammation and cytotoxic events, which can further cause BBB to be more extensive of leakage and destruction (Zenaro et al., 2017), see also Figure 1 for the transport and clearance of Aβ.

### Pathophysiological Functions of α-Synuclein in PD

Parkinson’s disease is the second most prevalent neurodegenerative disease, next only to AD. Amongst the diagnostic of more than 10 million people diagnosed with PD worldwide, the major clinic lesions are degenerative dopaminergic neurons in substantia nigra. Among 55–60-year-old populations, the incidence rate of PD is exponentially increased, imposing a heavy burden on the family and society (Baiano et al., 2020). The neurodegenerative disease is common in the aged, however, the onset of PD is not clear, in which risk factors are included but not limited to genetics, environment, as well as age. Unfortunately, no effective medications for the treatment of PD are available, where most of the current strategies are preventative medicine. Since the discovery of αSyn (*PARK1*) in the late 1990s, currently, there are 6 major causal risk genes (*SNCA*, *PRKN*, *UCHL1*, *PARK7*, *LRRK2*, and *PINK1*) that are associated with familial PD (Blauwendraat et al., 2020). However, only 5–10% of patients with PD have a family medical history, where most of them are sporadic.

Under physiological conditions, αSyn is a soluble protein that mainly localizes on vesicles in pre-synapse. Current understanding of the functions of αSyn is still limited to vesicle trafficking, recycling, and secretion. Clinic autopsy of brains with PD revealed inclusions of Lewy bodies and Lewy neurites in the ventral tegmental area (VTA) and substantia nigra (SN) in brainstem. Later, researchers gradually recognized that the major component of Lewy bodies and Lewy neurites are phosphorylated and misfolded αSyn, together with other proteins, like ubiquitin, neurofilament, and alpha B crystallin. Tau proteins may also be present. In 1996, for the first time, the scientist observed the ultrastructure of Lewy body by electron microscopy (Forno et al., 1996). Although the discovery of αSyn in PD was known to the public for decades, the mechanisms of its function, especially under pathological condition, remains largely unclear.

For many years, prion diseases were thought to be a unique group of neurodegenerative disorders in which the normal cellular prion protein (PrPc) was recruited by infectious prion protein through “seeded” fibrillization (Stohr et al., 2012). In recent decades, mounting evidence demonstrated that a prion-like self-propagating property may apply to a wide range of diseases with protein aggregation, including αSyn, tau, Aβ, huntingtin with polyglutamine (ployQ) repeats, superoxide dismutase 1 (SOD1), and TDP43. Pathological brain-derived lysates (αSyn, tau, and TDP43) were shown to act as templates or seeds that could efficiently recruit their soluble counterparts into insoluble fibrils in wild-type primary cultures, slice cultures, as well as *in vivo* (Ren et al., 2009; Chen et al., 2010; Munch et al., 2011; Volpicelli-Daley et al., 2011; Stohr et al., 2012; Iba et al., 2013).

Since human brains are valuable resources and are not available in every institute, people are seeking alternative options that could recruit pathological inclusions in wild-type tissues or *in vivo*. About 10 years ago, recombinant αSyn monomers were reported to spontaneously aggregate into the preformed fibrils (PFFs) and induce αSyn inclusions in wild-type primary cultured hippocampal neurons and *in vivo* (Volpicelli-Daley et al., 2011; Luk et al., 2012), as well as in slice cultures recently (Wu et al., 2020), which mimics the pathogenesis of PD in human brains. When self-template-dependent amplification for several rounds, these amyloidogenic PFF “seeds” not only recruit endogenous αSyn but also recruit tau into aggregates both *in vitro* and *in vivo* (Guo et al., 2013). In addition, under different salt conditions, these PFFs formed functionally and structurally different “strains” with distinct neurotoxicity and biochemical properties (Patterson et al., 2019). As for deceased patients diagnosed with PD, the glial cytoplasmic inclusions (GCIs) extracted from the cerebellum are 1,000 times more potent than αSyn PFFs in inducing pathology and are 100 times more potent than Lewy body extracted from PD with dementia (Peng et al., 2018). How and why the glial milieu modifies the β-sheet folding property of αSyn and contributes to its neuropathologic function opens for further investigation.

Considering the complexity and unpredictability of the structure of spontaneously formed αSyn PFFs, the potency of neurotoxicity varies from batches to batches, as this process was affected by salt, temperature as well as pH of the reaction system (Patterson et al., 2019). The biofluid samples (CSF and blood) from patients with AD and PD contain picogram levels of aggregated αSyn and tau, which can be used as template-dependent amplification seeds. One of the amplification techniques is the real-time quaking-induced conversion (RT-QuIC) assay, which can exponentially amplify a small amount of “seeds” from various biopsies with high sensitivity and specificity, making it a useful tool to aid in the clinical diagnosis of neurodegenerative disorders (Saijo et al., 2019). RT-QuIC was first successful in the diagnosis of human prion disease and then applied to detect αSyn aggregates in the brain and CSF from dementia with Lewy body and PD patients (Fairfoul et al., 2016). In the latest study, a group of RT-QuIC reactions for 4R tauopathies has been developed, specifically PSP, CBD, and FTDP-17 MAPT (Saijo et al., 2020). This emerging template-dependent amplification technique would benefit the diagnosis of AD and PD far before the onset of clinical lesions, and investigate the underlying mechanism of the structural and functional diversities of distinct brain-derived species is another challenging topic.

### Cell-Cell Transmission of Pathogenic “Seeds”

When adding PFFs into cultured hippocampal neurons, they compromised synaptic miniature EPSC firing frequency, inhibited spines formation, along with decreased action potential firing rate (Wu et al., 2019), where another group observed paradoxical functional defects induced by αSyn PFFs (Froula et al., 2018). On the other hand, the neuronal hyperactivity promoted the secretion of αSyn into extracellular space *in vivo* (Bassil et al., 2021) as well as *in vitro* (Bero et al., 2011), thus facilitated the propagation of pathology in CNS. These results were confirmed by an *ex vivo* slice culture system recently (Guo et al., 2013). The activity-dependent regulation of protein aggregation and spreading also applies to tau and Aβ in AD animal models (Wu et al., 2016; Bassil et al., 2021). For example, in a microdialysis study, neuronal activity modulated the concentration of Aβ and tau in the extracellular interstitial fluid of mouse brains (Bero et al., 2011). By imaging the traffic of fluorescent tag labeled tau in axons within microfluidic chambers, enhancing neuronal activity increased the release of tau and facilitated tau transmission (Wang et al., 2017). Furthermore, a study that used a chemogenetic approach to modulate neuronal activity demonstrated that hyperactivity leads to increase Aβ load and was reversed by inhibiting neuronal activity in a transgenic animal *in vivo* (Yuan and Grutzendler, 2016). Even before the formation of extracellular plaques, neurons in the prefrontal cortex showed hyperactivity and contributed to the progression of pathology *in vivo* (Zott et al., 2019), and this phenomenon was confirmed by *ex vivo* live imaging of hippocampal slice cultures in the 5xFAD model as well (Wu et al., 2020).

**FIGURE 2:**
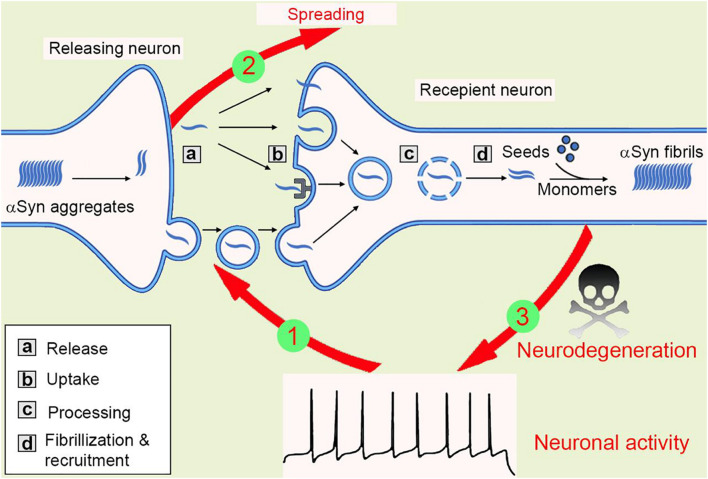
The spreading of αSyn aggregates between neurons. Mutations or neurotoxic environmental factors initialize the αSyn aggregation in the releasing neuron. The aggregates are released into extracellular via vesicle/exosome, and neuronal activity facilitates or inhibits this process (1). Then, the aggregates are taken up by macropinocytosis, LAG-3 receptor, or penetration into cell membrane directly. The aggregates transport inside the recipient cells, most of them will be degraded in lysosome, but some lysosomes will be ruptured and release aggregates, that are working as “seeds” to recruit endogenous αSyn monomers into new fibrils. Thus, the amyloidogenic aggregates spread between cells (2). The αSyn aggregates then compromise synaptic activity, inhibit spine formation, and finally result in neurodegeneration (3).

When zoom-in into the donor cells, the formation and amplification of pathological seeds initialize the transmission process. Both anterograde and retrograde transport of these proteins were observed, however, the Aβ fibrils were shown to be transported ten times more efficiently than αSyn (Freundt et al., 2012; Wu et al., 2013; Brahic et al., 2016). When released outside of the neurons, both tau and αSyn fibrils can be taken up via macropinocytosis, a form of endocytosis mediated by heparan sulfate proteoglycans (HSPGs) on the cell surface (Holmes et al., 2013). In another study, a pool of 352 complementary DNAs was expressed in SH-SY5Y cells and screened for αSyn-biotin PFF–binding candidates. This large-scale screening approach identified one of the top candidates, lymphocyte-activation gene 3 (LAG3), a transmembrane protein internalized via receptor-mediated endocytosis (Mao et al., 2016). After taken up through the plasma membrane, the fibrils then traffic along the endo-lysosomal pathway, process, rupture, and release from the lysosome into the cytosol before recruiting endogenous monomeric counterparts into insoluble inclusions (Figure 2), see also (Guo and Lee, 2014; Oueslati et al., 2014; Karpowicz et al., 2017). Moreover, tunneling nanotube (TNT)-mediated cell-cell transfer of pathological tau and αSyn seeds was observed *in vitro* recently (Abounit et al., 2016; Tardivel et al., 2016). However, whether TNTs connecting neurons are present *in vivo* remains unclear.

As for propagation and spreading of pathologies *in vivo*, the stereotypic progression of misfolded proteins in AD and PD follows a predictable pattern. Cross-sectional autopsy studies indicate that β-amyloid plaques first appear in the neocortex, followed by the allocortex and finally subcortical regions (Thal et al., 2002). Neurofibrillary tangles occur first in the locus coeruleus and transentorhinal area and then spread to the amygdala and interconnected neocortical brain regions (Braak and Del Tredici, 2011). Propagation of Lewy body/Lewy neurite in patients with PD is postulated to start in the brainstem and ascend toward neocortical regions with disease progression (Braak et al., 2003). The transmission of pathological tau and αSyn can occur bi-directionally within a network of interconnected populations, where the expression level of αSyn contributes to the selective vulnerability of hippocampal neuron subpopulations to fibrils-induced neurotoxicity (Luna et al., 2018).

### The Application of RNA Sequencing in Understanding Neuropathologies

Since the development of Tang’s single-cell RNA sequencing (scRNA-seq) method to understand the genomic transcriptome of mammalian cells in 2009 (Tang et al., 2009), this technique is booming as one of the most popular tools in the past decade. Previous scRNA-seq approaches from dissociated tissues use protease treatment at 30°C, which is known to alter the transcriptome. The brain is a complex mixture of neurons, glial and endothelial cells, making it hard to isolate the whole RNAs with traditional processes. Thus, with the development of the single nuclear RNA-seq (snRNA-seq) method, nuclei can be isolated at 4°C from tissue homogenates, which minimizes damage. Additionally, single nuclear transcriptomes can be obtained from postmortem human brain tissue stored at −80°C, making it possible to access the brain’s genetic “secrets” by RNA-seq via individual neurons. Here, we reviewed some of the recent progress of this emerging technology in the application of neurodegenerative diseases, and the latest spatial-temporal RNA-seq will also be discussed.

Poulin et al. (2016) summarized the quantitative analysis of markers in a single cell, enabling the classification of neural cells into categories (Please see Table 1 for more details and other recent literatures). With the understanding of molecular diversities of neurons via scRNA-seq, it’s getting closer to mapping the whole brain connectome at a single cell level. This will be an extremely important reference tool in the study of the nervous system and making unprecedented contributions to the field of neuroscience. Besides using it as the molecular classification tool, scRNA-seq has been largely applied in understanding mechanisms of diseases. By using microfluidic dynamic array-based scRNA-seq to simultaneously evaluate the expression of 96 genes in single neurons, Poulin et al. identified multiple novel subtypes of dopaminergic (DA) neurons which were localized in a distinct area of adult mouse brains. For example, Aldh1a1^+^ subtype DA neurons, located in the substantia nigra, are especially vulnerable in the 1-methyl-4-phenyl-1,2,3,6-tetrahydropyridine (MPTP) model of PD (Caudle et al., 2007).

**TABLE 1:** Summary of recent studies using RNA-seq in understanding AD/PD.

Author names	Year	Types of materials used for sequencing	The identified AD/PD-associated neurons or glial cells
Mathys et al.	2019	Frozen human tissues and identified major types of cells in the brain	Excitatory neurons (NRGN^+^), inhibitory neuron (GAD1^+^), astrocyte (AQP4^+^), oligodendrocyte (MBP), microglia (CSF1 and CD74^+^), oligodendrocyte progenitor cells (VCAN^+^), endothelial cells, and pericytes (AMBP^+^)
Jiang et al.	2020	Specific species (human or mouse), gender (male or female), brain region (entorhinal cortex, prefrontal cortex, or hippocampus, etc.)	MBP is the marker gene of oligodendrocyte cell type
Najm et al.	2020	Frozen mouse hippocampi containing the human neuron transplants at 7 MPT	The forebrain marker FOXG1, the dorsal telencephalic marker PAX6, and-to a lesser extent-the developing inhibitory neuron marker NKX2.1, neuronal markers MAP2 and TUJ1, as well as the cortical neuron marker TBR1, the excitatory neuron marker vGlut1, or the inhibitory neuron marker g-aminobutyric acid (GABA)
Zhong et al.	2020	Frozen mouse hippocampus	the four major subtypes of hippocampal neurons by interrogating the patterns of marker gene expression: DG neurons (marked by PROX1), CA1 neurons (marked by MPPED1), CA3 neurons (marked by MNDAL), and inhibitory neurons (marked by GAD1)
Wang et al.	2020	Identified from 263,370 single-cells in cortex samples by single-nucleus RNA sequencing (snRNA-seq) between 42 AD-pathology subjects and 39 normal controls	Up-regulated LINGO1 has been seen in both oligodendrocytes and excitatory neurons across 3 studies
Thrupp et al.	2020	Comparison of microglia from single cells and single nuclei of four human subjects	This population is enriched for genes previously implicated in microglial activation, including APOE, CST3, SPP1, and CD74
Aldaz et al.	2020	Specific neuron and interneuron subtypes from the cerebellum, frontal cortex, and hippocampus of mice	Neurons from the medial entorhinal cortex, Layer 5 from the frontal cortex as well as GABAergic basket cells and granule cells from cerebellar cortex are the specific neuronal subtypes that display the highest Wwox expression levels
Rosenzweig et al.	2019	The hippocampal DG in mouse	The homing macrophages expressed unique scavenger molecules including macrophage scavenger receptor 1 (MSR1)
Swanson et al.	2020	Formalin-fixed middle temporal gyrus (MTG) blocks from eight neurologically normal and eight AD cases from the Neurological Foundation Human Brain Bank	Iba1^low^ MOI^high^ myeloid cell populations delineated by MOIs CD45, HLA-DR, CD14, CD74, CD33, CD32, and L-Ferritin were increased in AD
Hook et al.	2018	Brain Tissue	Arcuate nucleus markers for Th^+^/Ghrh^–^ neuronal populations, e.g., Onecut2, Arx, Prlr, Slc6a3, and Sst
Kim et al.	2019	Induced pluripotent stem cells	Subpopulation 1 (SP1), subpopulation 2 (SP2) and subpopulation 3 (SP3) in the NSC neurospheres
Zhong et al.	2021	Cerebellum, midbrain, and striatum samples from mouse	GABAergic neuron (MB_GABA, ST_GABA, CB_GABA), glutamatergic neuron (MB_GLU, CB_GLU), serotonergic neuron (MB_SER), oligodendrocyte (MB_OLG, ST_OLG_1, ST_OLG_2, CB_OLG), oligodendrocyte precursor cell (MB_OPC, ST_OPC, CB_OPC), astrocyte (MB_AST, ST_AST_1, ST_AST_2, CB_AST), microglia (MB_MG, ST_MG_1, ST_MG_2, CB_MG), vascular cell (MB_VA), endothelial cell (ST_END), and pericyte (MB_PEC, ST_PEC, CB_PEC).
Grubman et al.	2019	Entorhinal cortex samples from control and Alzheimer’s disease brains	Transcription factor EB, a master regulator of lysosomal function, regulates multiple disease genes in a specific Alzheimer’s disease astrocyte subpopulation.
Selewa et al.	2020	All human tissues from a range of different samples, including tissues that are hard to dissociate, composed of fragile cells, and frozen specimens	Markers of CMs, including MYH6, TNNT2, MYL, and MYBPC3 and cardiac markers alongside markers of other lineages (e.g., FOXA2 and TTR)

The dysfunction of the striatum is associated with multiple neurodegenerative and cognitive diseases, including PD, hypersomnia, schizophrenia as well as addiction (De Deurwaerdere et al., 2017). According to the expression of D1 or D2 dopamine receptors, GABAnergic medium spiny neurons (MSNs) can be divided into D1 and D2 subtypes (Surmeier et al., 2007). By using SMART-seq2 of 1208 cells isolated from mouse striatum, the authors found that D1 and D2 MSNs can be further classified into subgroups. In addition, they also identified cell type-specific transcription and splicing factors, and these factors can change cell types by modulating transcription and gene expression profiles (Gokce et al., 2016). As a substantial neurotransmitter, dopamine plays an essential role in neurodegeneration and cognitive diseases (Caudle et al., 2007; Chen et al., 2008), thus, study the function of different types of dopaminergic neurons helps to understand the molecular basics and mechanisms of these diseases.

By analyzing single-cell transcriptomes of the GBA-N370S-PD iPSC-derived dopaminergic neurons, Lang et al. found a progression axis in response to endoplasmic reticulum (ER) stress. When analyzing differentially expressed genes (DEGs), the transcription inhibitor HDAC4 is upregulated during PD progression. In iPSC of PD patient-derived DA neurons, HDAC4 is mis-localized in the nuclear, inhibits gene expression during early progress of disease and results in defects of protein homeostasis in late-stage (Lang et al., 2019). In 2017, Ido Amit’s group discovered a new type of immune cells in the brain, disease-associated microglia (DAM), and proved that DAM is responsible to degrade dead cells and AD-related amyloid plaques. DAM is a conservative phagocytic cell in humans and mice, whose activation is dependent or independent on TREM2 (Keren-Shaul et al., 2017). The author emphasized that, only by performing scRNA-seq they can find these infrequent microglia, providing new strategies to treat AD and related disorders.

scRNA-seq has a high demand on the cell suspension viability and numbers prepared from organs or solid tissues, which means many frozen precious clinical samples (brain tissue, tumor tissue) is not applicable for this method. Thus, the snRNA-seq is another option that, to some extent, overcame this problem. As for brain tissues, scRNA-seq cannot fully analyze all types of neurons as some neurons are more easily to be affected by the dissociation process. Although the tolerance of non-neuronal cells from the neocortex of human adults is better than neurons during dissociation, more non-neuronal cells are left in single nuclear suspension. As for the neocortex of mouse brains, the proportions of layer-5 parvalbumin-positive neurons and glutaminergic neurons are lower than expected. In contrast, nuclear is more resistant to mechanical treatment and can be dissociated from frozen tissues. Single nuclear has been proofed to cover enough gene expression messages to define cell types of humans and the hippocampus of the mouse.

Previous research reported that single nuclear was isolated from frozen S1 cortex, followed with sorting with NeuN antigen, and then RNA sequenced with Fluidigm C1 system. Gene numbers and subtypes are comparable between nuclear and cells, with an average of 5,619 genes in S1 nuclear and an average of 4,797 genes in S1 cells, where nuclear has higher proportions of intron reads (Lake et al., 2018). Grubman et al. (2019) performed snRNA-seq of entorhinal cortex from deceased patients with Alzheimer’s disease and age-matched healthy controls and got 13,214 nuclear, with a median of 646 genes per cell. With AD autopsy cases, Mathys et al. (2019) performed snRNA-seq of frozen human tissues and identified major types of cells in the brain: excitatory neurons (NRGN^+^), inhibitory neuron (GAD1^+^), astrocyte (AQP4^+^), oligodendrocyte (MBP), microglia (CSF1^+^ and CD74^+^), oligodendrocyte progenitor cells (VCAN^+^), endothelial cells and pericytes (AMBP^+^).

The scRNA-seq technologies have enabled the use of single-cell transcriptional profiling to explore cellular heterogeneity. However, a common feature of scRNA-seq studies is that they do not resolve the spatial patterns or positional information, making it difficult to place cell-cell interactions within a broader tissue context. Harnessing *in situ* sequencing and quantitative analysis, the spatial transcriptomics (ST) refined these results and created a spatial subcellular map of 3D transcriptome profiles (Stahl et al., 2016; Salmen et al., 2018). In 2019, Asp et al. collected human embryonic heart tissue at different development stages (4.5–5, 6.5, and 9 post-conception weeks) and systematically analyzed subcellular resolution of regional markers and cell atlas based on ST and scRNA-seq (Asp et al., 2019). For example, in the AD mouse model, Chen et al. investigated the ST of 100 mm diameter around amyloid plaques and discovered early response genes that are enriched in myelin and oligodendrocytes, where plaque-induced genes (PIGs) involving the complement system, oxidative stress, lysosomes, and inflammation are prominent in the later phase of the disease (Chen et al., 2020). To improve spatial resolution, Slide-seq (Rodriques et al., 2019) and HDST (Vickovic et al., 2019) were developed, in which they used an alternative strategy with condensed beads-based technology, making it feasible to profile genome-wide molecular events at the single-cell level.

Overall, snRNA-seq is more universally applicable than scRNA-seq, it does not limit to fresh tissue, but also frozen ones. Additionally, a single nucleus is easier to prepare than a single cell because it requires less enzymatic digestion, thereby reducing pseudo-cell subtypes induced by mechanical stress. In terms of data analysis, snRNA-seq contains information about introns and intergenic regions, making the resolution of cell subtypes higher. SnRNA-seq cannot replace scRNA-seq. The choice of RNA-seq method depends largely on experimental conditions, such as sample, cell suspension, enzyme sensitivity and other factors. The combination uses of both scRNA-seq and snRNA-seq would be the ideal strategy to get full access to genome transcriptions.

## Conclusion

The accumulation of αSyn aggregates in Parkinson’s and related diseases is unstoppable, and preclinical symptoms can occur about 10–20 years before the lesions of neuronal functions. Though people have developed multiple strategies to predict, investigate and slow down the process, the progression is far behind expected. Biochemistry, molecular biology, autopsy, electron microscopy, and live imaging techniques enable us to learn the underlying mechanism of protein aggregation and spreading between neurons, however, all these traditional assays are biased focused on one or two genes at a time and neglect the complexity and integrity of the biological system. In the last decade, the booming of scRNA-seq and snRNA-seq techniques lead us into a “big data” era that tens of thousands of genes and signal pathways have been uncovered within the same tissues. These big data also bring us too much “noise” and it should be more careful to choose what kind of pathways or genes that fit your hypothesis in the future. And how to use the published database to explore useful information is another recycling of resources, which, of course, will benefit and guide the preclinical research.

## Author Contributions

JJ and YL wrote and edited the manuscript. QW designed, conceived, wrote, and edited the manuscript. All authors approved of the final manuscript.

## Conflict of Interest

The authors declare that the research was conducted in the absence of any commercial or financial relationships that could be construed as a potential conflict of interest.

## Publisher’s Note

All claims expressed in this article are solely those of the authors and do not necessarily represent those of their affiliated organizations, or those of the publisher, the editors and the reviewers. Any product that may be evaluated in this article, or claim that may be made by its manufacturer, is not guaranteed or endorsed by the publisher.
